# Development of *Synthesiomyia nudiseta* (Diptera: Muscidae) Under Constant and Fluctuating Temperatures: Implications for Minimum Postmortem Interval Estimation

**DOI:** 10.3390/insects17100982

**Published:** 2026-09-23

**Authors:** Ren Long, Chengyong Luo, Yuting Xie, Ziling Guo, Chenxi Liu, Ziheng Xu, Gaolin Zheng, Tao Song

**Affiliations:** 1 Key Laboratory of Tropical Translational Medicine of Ministry of Education, School of Basic Medical Sciences, Hainan Academy of Medical Sciences, Hainan Medical University, Haikou 571199, China; hy0206055@muhn.edu.cn (R.L.); yuting@muhn.edu.cn (Y.X.); guoziling0301@163.com (Z.G.); 0817@muhn.edu.cn (C.L.); 2054762833@muhn.edu.cn (Z.X.); zhenggaolin0602@gmail.com (G.Z.); 2 Guangdong Nantian Institute of Forensic Science, Shenzhen 518033, China; lcy@nantian.org

**Keywords:** muscid fly, *Synthesiomyia nudiseta*, forensic entomology, development model, fluctuating temperature

## Abstract

*Synthesiomyia nudiseta* (van der Wulp, 1883) is widely distributed across pantropical and subtropical biogeographic regions. Despite its recognized forensic importance, comprehensive developmental data for it remains scarce. This study observed the development of *S. nudiseta* from egg to adult at six constant temperatures in the range of 20–35 °C, and found that the times required were 732.7 ± 7, 587.3 ± 4.7, 449 ± 21.3, 393.7 ± 5.7, 361.3 ± 8.3 h, respectively. It cannot complete development at 35 °C. We established three development models to estimate the age of the immature insect: isomegalen diagram, isomorphen diagram, and thermal summation model. A regression analysis was performed on the relationship between body length and total development time from hatching to pupariation. The thermal summation constant during the development of *S. nudiseta* was 7870.1 degree-hours, and the developmental threshold temperature was 9.28 °C. In addition, we observed development of *S. nudiseta* under four fluctuating temperature regimes; developmental duration exhibited a negative correlation with increasing mean temperature; however, inclusion of 35 °C within the fluctuating temperature cycle prevented successful adult eclosion, with the vast majority of pupae succumbing to mortality before reaching the adult stage. The results of this experiment provide a basis for the use of *S. nudiseta* in the estimation of PMI_min_.

## Simple Summary

*Synthesiomyia nudiseta* is a common insect that helps estimate the time since death in forensic cases. Despite its recognized forensic importance, comprehensive developmental data for it remains scarce. In this study, we observed the development of *S. nudiseta* from egg to adult at six constant temperatures and four fluctuating temperature regimes, and we established three development models to estimate the age of the immature insect: isomegalen diagram, isomorphen diagram, and thermal summation model. We found that it cannot complete development at 35 °C, and FT4 cannot complete the full developmental cycle. This work improves the scientific basis for using *S. nudiseta* to estimate death time, supports more reliable forensic investigations, and helps promote justice in criminal cases.

## 1. Introduction

Forensic entomology provides biologically rigorous and reliable postmortem interval (PMI) estimates by analyzing the succession and developmental patterns of necrophagous insects colonizing human remains [1,2]. A core methodological approach in this discipline is to estimate the minimum postmortem interval (PMI_min_) based on the age of the oldest immature stages of necrophagous insects recovered from corpses [3,4,5]. The concept of PMI_min_ was established to overcome uncertainties surrounding the pre-colonization interval of necrophagous insects, given the absence of conclusive scientific evidence defining their precise arrival time on decomposing remains. The accuracy of PMI_min_ estimation inherently depends on the availability of precise species-specific developmental data obtained from controlled laboratory experiments [6,7,8].

*Synthesiomyia nudiseta* (van der Wulp, 1883) is the sole recognized species of the genus *Synthesiomyia* within the family Muscidae. Natively distributed across pantropical and subtropical biogeographic regions worldwide, it has successfully invaded and established persistent populations in Southern Europe in recent decades, driven by global trade and climate change, and has emerged as a key species with rapidly growing attention in cross-regional forensic entomological investigations [9,10,11]. As a species with a pronounced synanthropic lifestyle, its activity range closely overlaps with human residential and activity environments, which significantly increases its likelihood of being recovered in human death investigations [9]. This species is frequently collected from human cadavers and is well-documented as one of the dominant necrophagous fly species in indoor, enclosed, and concealed crime scenes, as well as in cadavers retrieved from urban settings [12,13,14]. In a systematic forensic case survey conducted in Taiwan, China, this species exhibited a 36% detection rate in cases involving indoor cadavers and a 41% detection rate in cadavers from urban environments, rendering it the most prevalent forensic insect species in indoor death cases [15]. Meanwhile, this species is consistently recovered from cadavers across the advanced decomposition to skeletonization stages, and can also co-occur with first-wave colonizing taxa including Calliphoridae and Sarcophagidae in cases where it colonizes cadavers during the early post-mortem phase. This unique biological attribute confers irreplaceable application value to the species in scenarios requiring the estimation of long post-mortem intervals [16].

Although *S. nudiseta* is widely acknowledged to hold considerable forensic entomological value, systematic and complete developmental data for this species are still relatively limited. Previous studies have offered valuable preliminary insights into its life cycle and developmental characteristics, yet most available research remains somewhat fragmented and is largely confined to a narrow range of constant temperature regimes [17,18]. In addition, in practical forensic case investigations, insects develop under naturally fluctuating thermal conditions, instead of the constant temperature settings commonly adopted in most laboratory experiments. Some studies have indicated that insect developmental rates under moderately fluctuating temperatures are basically consistent with those observed under corresponding constant mean temperatures [19,20]. However, remarkable deviations in developmental progression tend to occur when ambient temperatures approach the lower or upper critical thermal limits of a given insect species [21]. Some research under fluctuating temperatures showed that different necrophagous blow flies had diverse developmental responses, including accelerated development and delayed development [19,21,22,23]. Therefore, it is necessary to incorporate both constant temperatures and ecologically realistic fluctuating temperature regimens into laboratory experiments, so as to improve the practical applicability of developmental data in forensic casework.

The present study aimed to systematically characterize the developmental biology of *S. nudiseta*. This species was reared at six constant temperatures, as well as under four ecologically relevant fluctuating temperature regimes that simulate local stable occurrence environments of this species. Key developmental parameters evaluated included stage-specific developmental duration, accumulated degree hours (ADH), and larval body changes. Based on the obtained data, forensic predictive models, including isomorphen diagrams, isomegalen diagrams, and thermal summation models, were established to support PMI_min_ estimation in forensic cases associated with *S. nudiseta*.

## 2. Materials and Methods

### 2.1. Colony Establishment

In March 2025, approximately 20 adult *S. nudiseta* individuals (1:1 male-to-female ratio) were collected from fresh pig lung bait placed outdoors at Hainan Medical University, Haikou, Hainan Province, China (20°2′48.7″ N, 110°11′44.4″ E). Field-collected adults were used to establish a laboratory colony, and species identification was performed using adult morphological taxonomic keys [24].

Adults were reared in a nylon cage (35 × 35 × 30 cm) maintained at ambient room temperature (approximately 26 °C, 75% relative humidity (RH)) under a natural photoperiod. Adults were provided ad libitum with a 1:1 mixture of milk powder and granulated sugar as food, along with fresh water. To induce oviposition, a 15 cm-diameter Petri dish baited with fresh pig lung tissue was placed inside the rearing cage. After oviposition, the Petri dish containing pig lung tissue and egg masses was removed from the cage and transferred to a ventilated rearing container fitted with a nylon mesh lid. The bottom of each container was lined with a 2–3 cm layer of moist sandy soil to provide a suitable pupariation substrate for post-feeding larvae. The containers were then placed in an artificial climate incubator (Model RXZ-160A, Jiangnan Co., Ltd., Ningbo, China) and incubated under controlled conditions of 26 °C, 75% relative humidity (RH), and a 12:12 h light/dark (L/D) photoperiod until adult eclosion. Upon eclosion, the container lids were opened, and newly emerged adults were released into the main breeding cage to replenish the laboratory colony for subsequent thermal development assays. Throughout the experimental period, the adult population in the colony was kept stable at 1000–1500 individuals.

### 2.2. Development Duration Observation and Larval Sampling

To induce oviposition for subsequent experiments, approximately 20 g of fresh pig lung tissue was placed in a 15 cm diameter Petri dish, which was then transferred into the laboratory-rearing cage. Eggs deposited within a 2 h period were collected to ensure developmental synchrony. A total of 600–800 eggs were equally allocated into two 15 cm diameter Petri dishes, serving as one biological replicate under a given temperature regime; this arrangement prevented temperature elevation resulting from larval overcrowding. Each dish was supplemented with an additional 20 g of fresh pig lung tissue as larval food, and then placed into a rearing box lined with 2–3 cm of moist sandy soil. Generally, fresh pig lung (approximately 20 g each time) was added 1–3 times per day according to larval feeding status. Fresh pig lung was no longer added when half of the larvae started dispersing.

All rearing boxes were placed in programmable micro-environment incubators (model RXZ-160A, Jiangnan Co., Ltd., Ningbo, China). Relative humidity was set constant at 75% throughout the experiment, with a 12:12 h light/dark photoperiod. Treatments comprised six constant-temperature conditions (20 °C, 23 °C, 26 °C, 29 °C, 32 °C, and 35 °C) and four fluctuating-temperature regimes simulating natural thermal variation within the native range of *S. nudiseta*. The fluctuating temperature treatments were designated as FT1 to FT4: FT1 (mean = 23 °C; sequential temperature phases: 20 °C, 23 °C, 26 °C), FT2 (mean = 26 °C; sequential temperature phases: 23 °C, 26 °C, 29 °C), FT3 (mean = 29 °C; sequential temperature phases: 26 °C, 29 °C, 32 °C), and FT4 (mean = 32 °C; sequential temperature phases: 29 °C, 32 °C, 35 °C). Each temperature phase within a single fluctuation cycle lasted 8 h. 

Referring to the developmental timeline determined in preliminary trials, eggs were examined at 1 h intervals starting 3 h before the predicted hatching time to record the precise hatching moment. After hatching, six individuals (three larvae from each of the two Petri dishes) were collected at 8 h intervals until the onset of pupariation. During larval rearing, around 20 g of fresh pig lung tissue was supplied to each Petri dish once or twice daily according to larval feeding consumption, to ensure ad libitum food availability and avoid starvation. The sampled larvae were instantly killed by immersion in hot water at 90 °C for 30 s and then preserved in 80% ethanol for subsequent morphological analysis. Larval instar was identified under a Zeiss 2000-C stereomicroscope (Carl Zeiss AG, Oberkochen, Germany) based on morphological characteristics of the posterior spiracle. Key developmental events, including egg hatching, pupariation, and adult eclosion, were monitored and recorded at 8 h intervals throughout the experiment. Larval body length was measured using a digital vernier caliper (precision: 0.01 mm; Shengong, Shanghai, China), with measurements taken from the anterior apex of the cephalopharyngeal skeleton to the posterior tip of the abdomen. All constant and fluctuating temperature treatments were each conducted in three independent experimental replicates using separate incubators to account for methodological variability.

### 2.3. Statistical Analysis

All statistical analyses were performed using Origin Pro 2024b (OriginLab Corporation, Northampton, MA, USA). An isomorphen diagram was constructed using developmental duration data for each key developmental event, with the *x*-axis representing developmental time (hours) and the *y*-axis representing constant experimental temperature (°C).

The revised linear regression model proposed by Ikemoto and Takai [25] was utilized to conduct linear regression analysis on the relationship between developmental time and accumulated degree hours (ADH) across all key developmental stages. The lower developmental thermal threshold temperature (TL) and the thermal summation constant (K) of *S. nudiseta* were derived from the slope and intercept of the resulting linear regression equations, respectively.

A scatter plot was generated to visualize changes in larval body length of *S. nudiseta* from hatching to pupariation, with “time after hatching” (days) as the independent variable and “larval body length” (mm) as the dependent variable. Polynomial regression analysis was conducted to model the relationship between larval body length and time after hatching, with the primary objective of establishing predictive equations for the estimation of PMI_min_ in forensic casework. An isomegalen diagram was plotted using the data calculated from these polynomial regression equations to enable visual estimation of larval age from body length measurements at different temperatures.

A one-way ANOVA + Tukey test was used to verify the influence of temperature conditions (constant-temperature vs. fluctuating-temperature) on the developmental durations (*p* < 0.05).

## 3. Results

### 3.1. Developmental Duration and Construction of the Isomorphen Diagram

*Synthesiomyia nudiseta* successfully completed its entire developmental cycle from egg hatching to adult eclosion across the constant temperature range of 20–32 °C. In contrast, at a constant temperature of 35 °C, the species was unable to complete egg hatching, with all eggs failing to develop to the larval stage. The duration of key developmental events (hatching, first ecdysis, second ecdysis, pupariation, and eclosion) for *S. nudiseta* under constant temperatures decreased significantly with increasing temperature, from a total developmental time of 732.7 h at 20 °C to 361.3 h at 32 °C (Table 1).

An isomorphon diagram was constructed based on the time required to reach each developmental event at different constant temperatures (*x*-axis: developmental time; *y*-axis: temperature) (Figure 1). Within the 20–32 °C constant temperature range, the elapsed time between successive developmental events (hatching, first ecdysis, second ecdysis, pupariation, and eclosion) gradually decreased with increasing temperature, and the spatial distance between the curves representing each developmental event on the diagram was correspondingly reduced, reflecting the accelerated developmental rate at higher temperatures.

### 3.2. Thermal Summation Model

Linear regression analysis was performed to calculate the developmental thermal threshold temperature (TL) and the thermal summation constant (K) of *S. nudiseta* for each key developmental stage and for the total developmental cycle (egg to adult eclosion) (Figure 2).

The coefficient of determination (R^2^) for all linear regression models exceeded 0.96, indicating a high degree of fit between the experimental data and the linear regression model. For the total developmental process of *S. nudiseta*, the TL was calculated as 9.28 ± 1.03 °C, and the K was 7870.1 ± 538.5 degree hours (Table 2).

### 3.3. Change in Larval Body Length and Construction of the Isomegalen Diagram

Polynomial regression analysis was conducted with “time after hatching” as the independent variable and “larval body length” as the dependent variable to model larval growth dynamics (Figure 3), and predictive equations describing changes in body length over time under different constant temperatures were generated (Table 3).

An isomegalen diagram was constructed using the developmental data derived from the polynomial regression models (Figure 4), enabling the estimation of larval age (time after hatching) from measured body length at any temperature within the 20–32 °C range.

### 3.4. Comparison of Developmental Duration over Fluctuating and Constant Temperatures

Four fluctuating-temperature treatments (FT1–FT4) had mean temperatures of 23 °C, 26 °C, 29 °C, and 32 °C, which matched four constant-temperature regimes used in this experiment. The total developmental durations of *S. nudiseta* were measured under each constant temperature and its corresponding fluctuating-temperature regime (Table 1 and Table 4). For FT1, FT2, and FT3, the developmental durations across developmental stages were close to those under their matched constant temperatures of 23 °C, 26 °C, and 29 °C, respectively. No significant differences in stage-specific developmental durations were detected between each fluctuating-temperature regime and its paired constant-temperature counterpart (*p* > 0.05). In contrast, under FT4 with a mean temperature of 32 °C, although early-stage developmental durations were comparable to those at the constant 32 °C and showed no statistical difference, *S. nudiseta* failed to emerge as adults under this fluctuating-temperature condition (Figure 5).

## 4. Discussion

Comparisons with published developmental literature on *S. nudiseta* show that our findings are consistent with some previous reports but differ greatly from others (Table 5). At a constant temperature of 20 °C, the total developmental duration measured in this study was 30.5 d. This value was close to 26.33 d reported from Alicante, Spain [10], with a difference of 4 d, but considerably longer than 44.9 ± 25.5 d reported from Caracas, Venezuela [17]. At 26 °C and 29 °C, the total developmental durations from oviposition to adult eclosion were 18.7 d and 16.4 d, respectively. These values were highly consistent with 17.50 d and 15.39 d obtained by Velásquez et al. [10] at the nearby temperatures of 25 °C and 30 °C. However, at 26 °C, Krüger et al. [26] and Aruna Devi et al. [27] reported developmental durations that were 9 d longer and 4.7 d shorter than ours. Kumara et al. [28] recorded a duration of 13.4 d under 28.5 ± 1.5 °C, whereas our result was 16.4 d at constant 29 °C, 3 d longer than their value. Rabinovich [17] reported 32.7 d at 28 ± 1 °C, which was 16.3 d longer than our result. These results indicate that developmental data are comparable among some studies under the same or similar temperatures, while obvious discrepancies also exist between other reports.

Several factors have been suggested to explain such discrepancies, including biological variables and experimental methods, such as geographic origin of experimental populations, larval food sources, and rearing conditions [29,30,31,32]. Populations of the same fly species from different geographic regions show certain differences in genetic background, and genetic variation can partly lead to divergence in developmental duration [32].

In addition, larval food source represents an important source of variation among published datasets. Most previous studies used beef or pork liver as larval diet, while fresh pig lung tissue was used in the present experiment. It has been confirmed that food source can modify developmental rates of necrophagous flies, and this factor may largely account for inconsistent results among *S. nudiseta* studies [30]. Studies on other forensically important fly species have demonstrated that larval food type directly affects growth rate and developmental duration. For instance, Clark et al. [31] found that *Lucilia sericata* larvae grew faster on pig-derived tissues than on cattle-derived tissues; larvae fed lung and heart tissue developed more rapidly than those reared on liver. Kaneshrajah and Turner [33] also reported that *Calliphora vicina* larvae fed porcine lung, kidney, heart and brain tissue completed development 2 d earlier than those raised on porcine liver. These findings suggest that tissue type strongly influences larval fly development, and may explain the differences between our results (pig lung) and earlier studies on *S. nudiseta* (liver or beef) [10,27].

Apart from food sources, rearing conditions can also produce data variation. Humidity is a critical variable in developmental experiments. High humidity causes larvae to crawl extensively inside rearing containers and delays pupariation of post-feeding larvae. Low humidity dries meat food, prevents larval feeding, and slows development. Previous work has also shown that photoperiod can alter insect developmental progress [34].

In this study, temperature, humidity, and light conditions were strictly controlled to minimize experimental interference with the developmental rate of *S. nudiseta*.

Using the modified linear regression model proposed by Ikemoto and Takai [25], the lower developmental threshold of *S. nudiseta* was calculated as 9.28 °C, about 2 °C higher than the value of 7.3 °C reported by Velásquez et al. [10]. Since model outputs are predictive estimates, the true low-temperature threshold of this species still needs further verification. In contrast, we investigated its high-temperature tolerance threshold experimentally. To our knowledge, this is the first study to document developmental durations of *S. nudiseta* at temperatures ≥32 °C, filling data gaps under high-temperature conditions. Our results show that *S. nudiseta* can complete full development at a constant 32 °C, which represents the upper viable constant temperature under our experimental conditions. 

Constant 35 °C is lethal to eggs, and no eggs hatched at this temperature. Even under fluctuating-temperature regimes, survival of eggs and pupae decreased sharply whenever 35 °C was included in the thermal cycle. For example, *S. nudiseta* can complete the whole life cycle from egg to adult at a constant 32 °C. Nevertheless, under FT4 with a mean temperature of 32 °C, partial egg mortality and complete pupal mortality were observed. This can be mainly attributed to the inclusion of 35 °C in the FT4 thermal cycle, which exerts strong lethal effects on eggs and pupae. Collectively, these results suggest that 35 °C exceeds the physiological upper thermal limit for eggs and pupae of *S. nudiseta*. High temperatures induce multiple physiological stresses in necrophagous flies, including egg dehydration, abnormal embryonic development, and metabolic disorders during the pupal stage; these factors may contribute to the high mortality observed at 35 °C. Our study implies that *S. nudiseta* is not a suitable indicator for PMI_min_ estimation in hot environments where ambient temperatures frequently approach or exceed 35 °C. For such high-temperature case scenarios, forensic entomologists should prefer heat-tolerant necrophagous fly species such as *Chrysomya megacephala*. This blow fly exhibits strong heat resistance and is widely applied for PMI_min_ estimation in tropical and subtropical regions.

Isomorphen and isomegalen diagrams offer convenient tools for rapid PMI_min_ estimation in case investigations. However, these models are only valid under conditions with stable mean temperatures [35]. When using isomegalen diagrams to estimate larval age, it is necessary to distinguish feeding-stage larvae from wandering-stage larvae. Body-length data used in isomegalen diagrams correspond to development from hatching to peak feeding, and cannot be applied after larvae begin body shrinkage [36]. In forensic entomology practice, developmental data obtained under constant temperatures are commonly used for PMI_min_ estimation. Investigators measure the average temperature at the corpse site and apply corresponding constant-temperature developmental datasets [4]. Field temperatures at crime scenes are inherently dynamic and fluctuating rather than constant. Therefore, theoretically, the optimal strategy for PMI_min_ estimation is to use developmental data generated under fluctuating temperatures that match the actual thermal environment experienced by insects on corpses.

Many studies have compared insect development under fluctuating versus constant temperatures and reached two different conclusions. Some studies report minor effects of temperature fluctuation on developmental rate, while others show significant influences on insect development and survival [37]. Such inconsistent conclusions may result from differences in fluctuation amplitude, mean temperature, and species-specific thermal tolerance. In the present study, standardized fluctuating-temperature regimes were set around the mean temperature with a fixed amplitude of ±3 °C; each thermal phase lasted 8 h. Our results indicate that moderate temperature fluctuation of this type does not significantly alter the developmental rate of *S. nudiseta*. Larval growth and developmental durations were comparable to those observed under corresponding constant mean temperatures. This finding agrees with previous reports on other necrophagous flies: within a species’ viable thermal range, moderate temperature fluctuation does not change developmental rates relative to the constant mean temperature [38].

## 5. Conclusions

This study systematically characterized the complete developmental profile of *S. nudiseta* under six constant-temperature treatments (20–35 °C) and four ecologically relevant fluctuating-temperature regimes. Three practical forensic prediction tools, namely the isomorphen diagram, isomegalen diagram, and thermal summation model, were successfully established for estimating the age of immature *S. nudiseta*. Under constant thermal conditions, this species could complete its full life cycle from egg to adult eclosion across 20–32 °C, yet constant 35 °C was lethal to eggs and prevented hatching entirely. The calculated lower developmental threshold temperature for the whole developmental cycle was 9.28 °C, with a thermal summation constant of 7870.1 degree hours. For fluctuating-temperature groups with mean temperatures of 23 °C, 26 °C, and 29 °C, no statistically significant differences in developmental duration were detected compared with their corresponding constant-temperature counterparts, implying that moderate temperature fluctuation within the viable thermal range does not alter the developmental rate of *S. nudiseta.* Nevertheless, the fluctuating regime FT4 that incorporated periodic 35 °C exposure caused complete pupal mortality and failed adult emergence, even though its mean temperature was 32 °C, demonstrating that transient exposure to 35 °C exceeds the upper physiological thermal limit for eggs and pupae of this species. Our findings supply species-specific developmental baseline data for PMI_min_ estimation using *S. nudiseta*. Forensic practitioners should be alerted that *S. nudiseta* may not be suitable for casework in persistently hot environments where temperature frequently reaches 35 °C. Further field validation is still required to evaluate the real-world applicability of these laboratory-derived developmental models under complex crime-scene conditions.

## Figures and Tables

**Figure 1:**
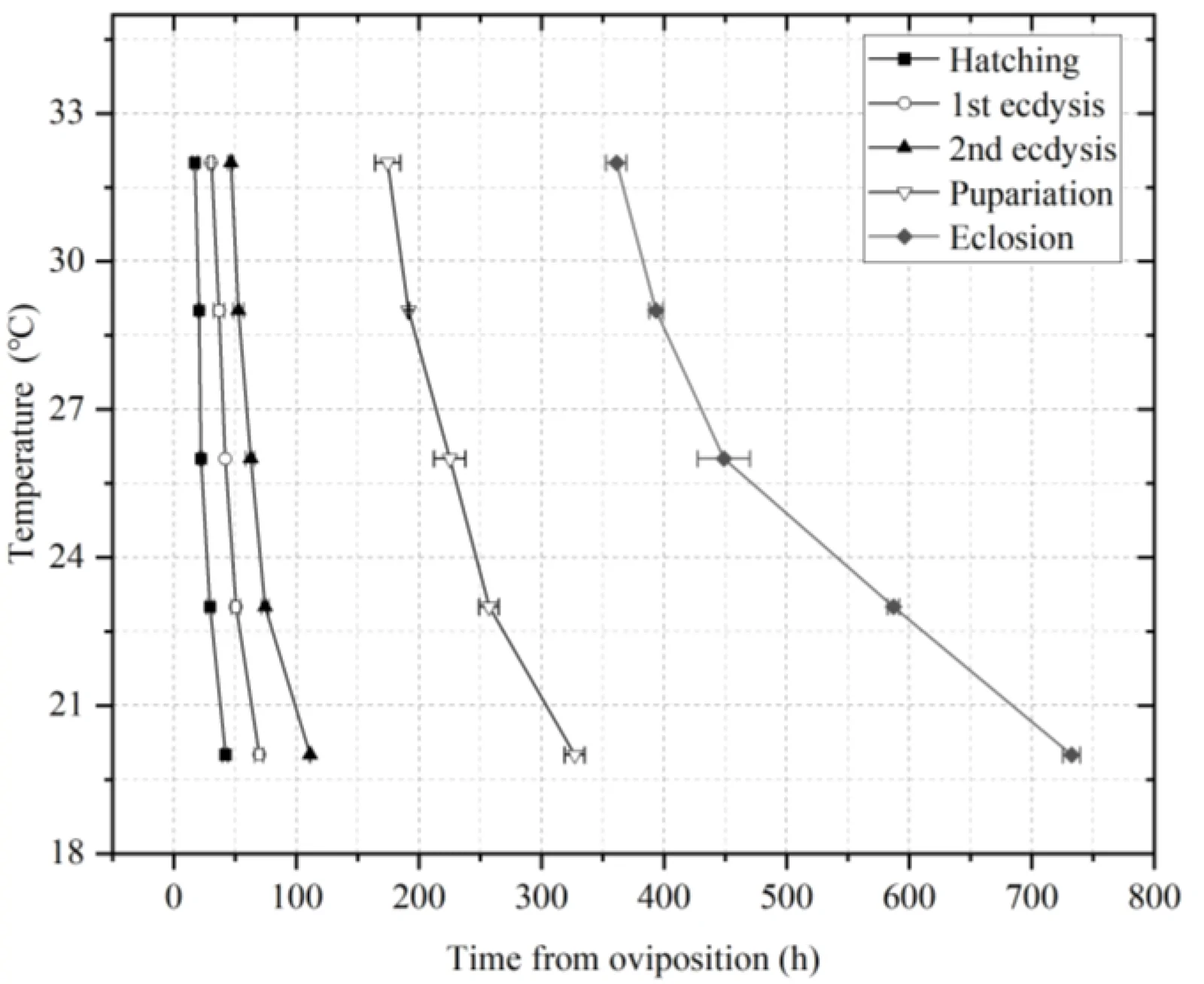
Isomorphen diagram of *S. nudiseta*. The duration of each developmental event (hatching, first ecdysis, second ecdysis, pupariation, and eclosion) is plotted against the time from oviposition to the onset of each event. Each curve corresponds to a particular development event. The error bar represents the SD.

**Figure 2:**
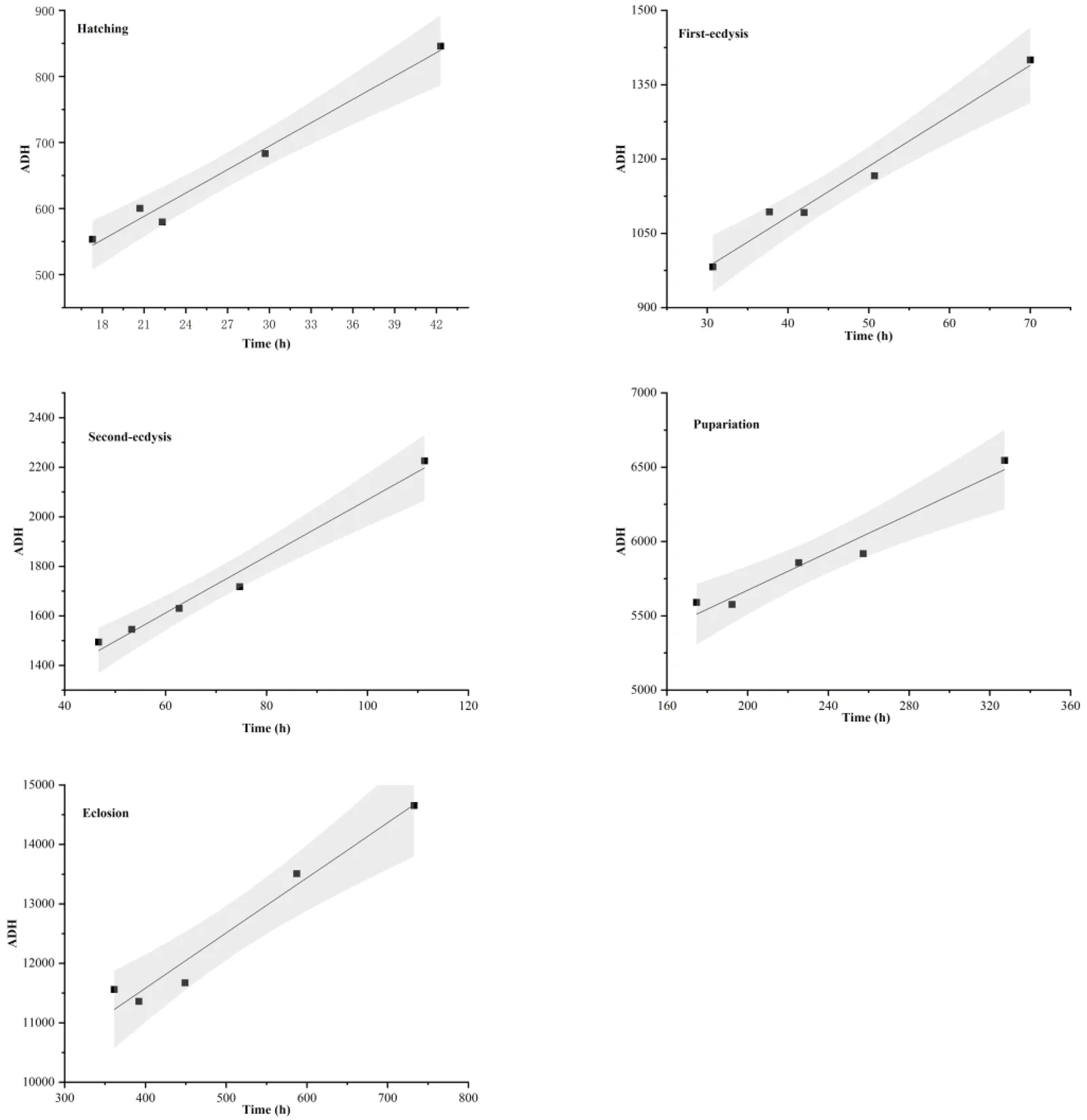
Thermal summation models of three developmental stages and total development duration of *S. nudiseta*. “▪” denotes data used in the regression analysis. The solid line represents the regression line. The gray shadow represents the 95% confidence interval.

**Figure 3:**
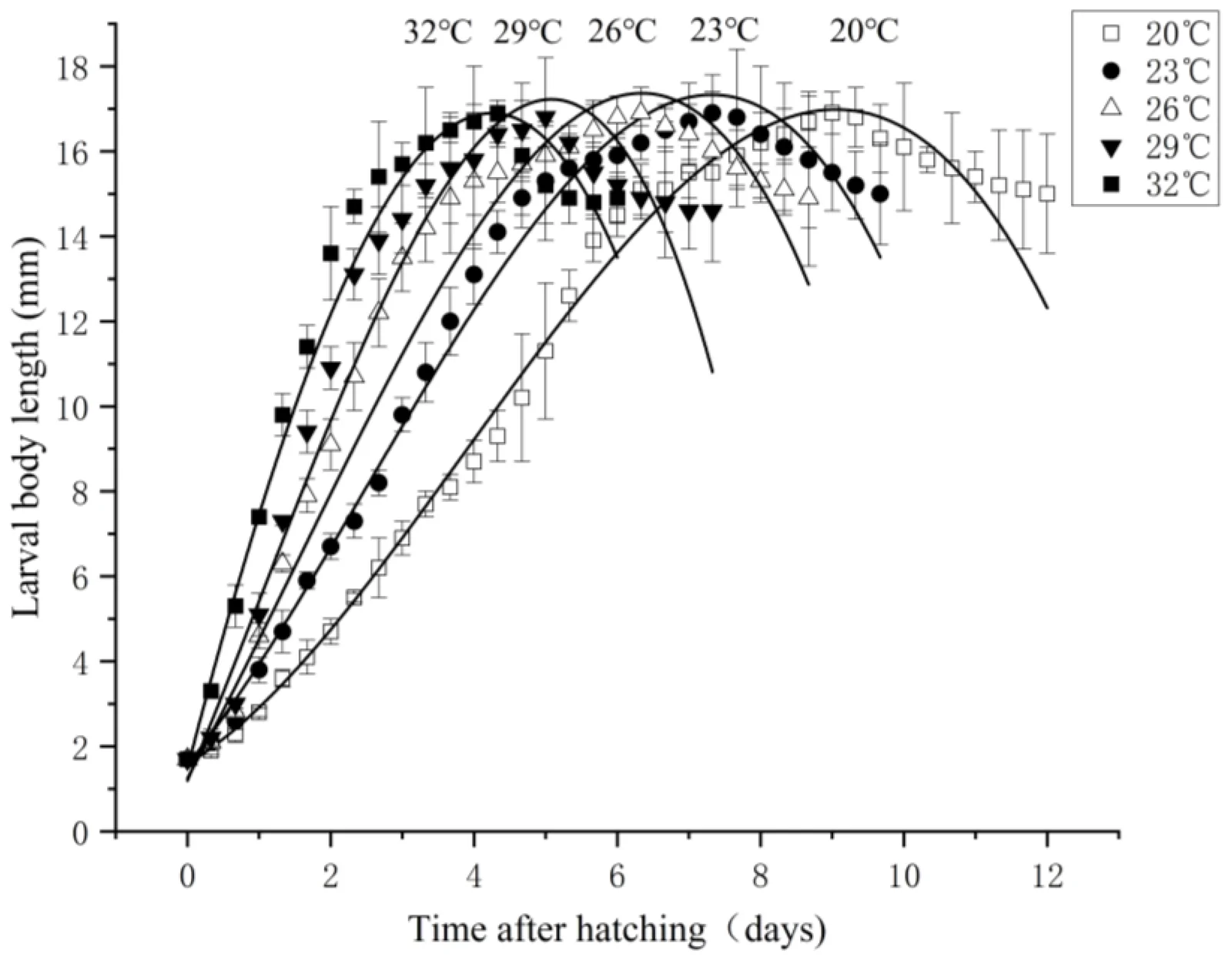
Larval body length changes in *S. nudiseta* under different temperature conditions.

**Figure 4:**
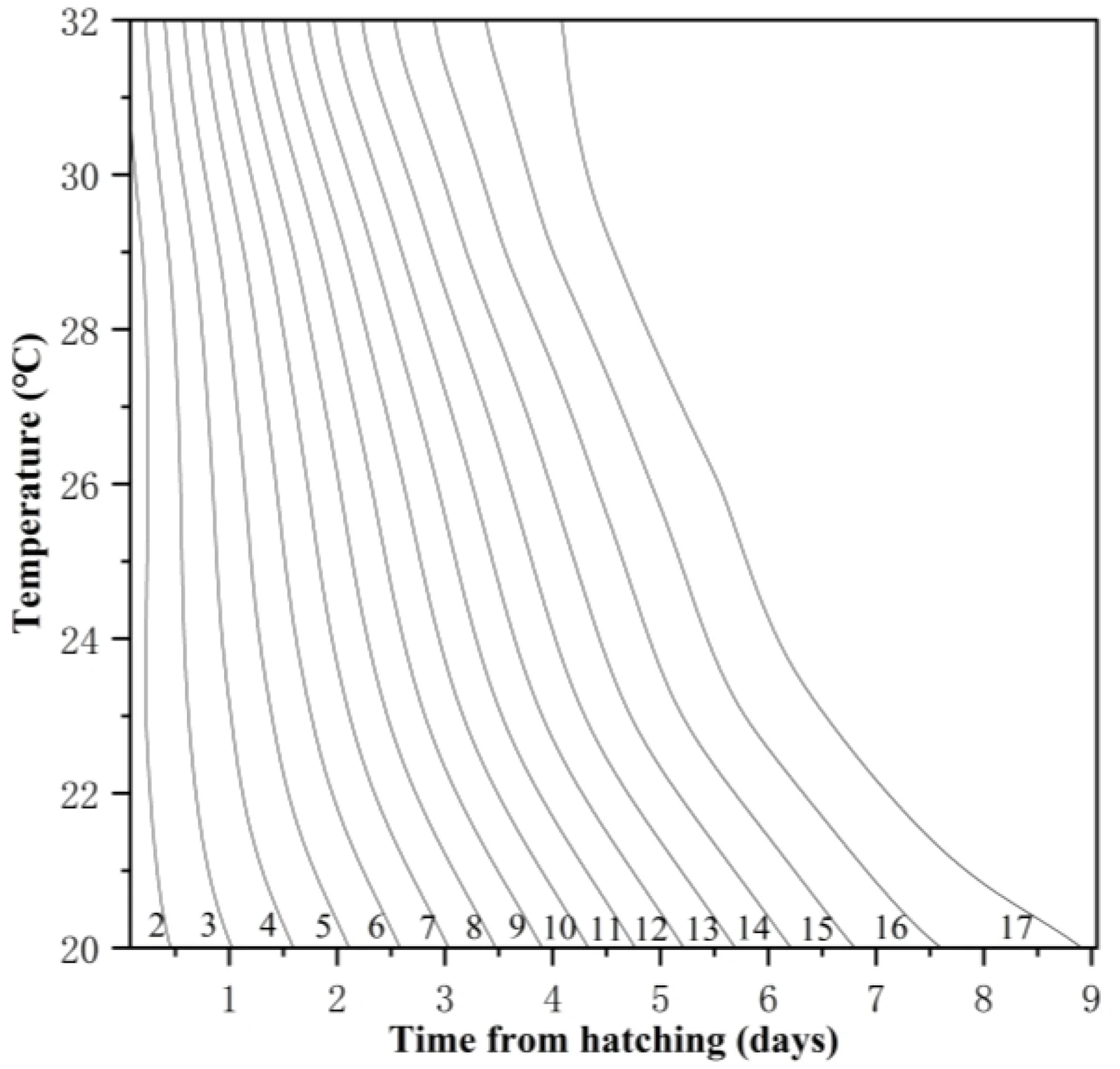
Isomegalen diagram of *S. nudiseta*. Each contour represents larval length in relation to their age (*x*-axis) and the temperature experienced during development (*y*-axis). The dimension of the length is not needed visually to calculate a PMI_min_; therefore, it is not illustrated.

**Figure 5:**
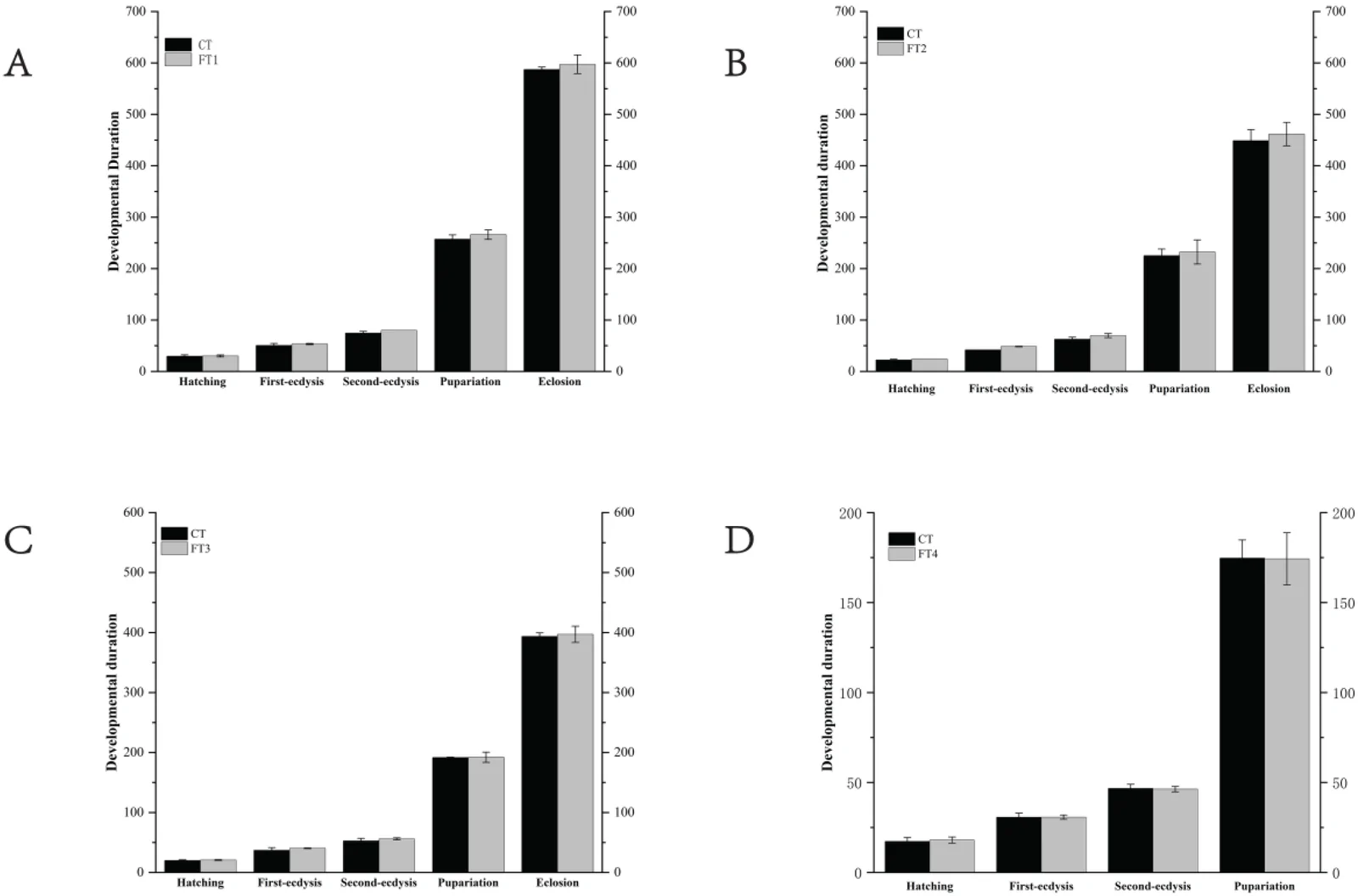
Comparison of developmental durations of *S. nudiseta* under different temperature regimes at four gradients. (**A**), (**B**), (**C**) and (**D**) indicate the developmental durations of 23 °C, 26 °C, 29 °C, and 32 °C gradients, respectively. Gray and black bars indicate FT and CT, respectively.

**Table 1:** Mean (±SD) time (h) to developmental event of *S. nudiseta* at six constant temperatures.

Temperature (°C)	Hatching	First-Ecdysis	Second-Ecdysis	Pupariation	Eclosion
20	42.3 ± 2.1	70 ± 3.5	111.3 ± 7.6	327.3 ± 8.1	732.7 ± 7.0
23	29.7 ± 2.5	50.7 ± 3.8	74.7 ± 3.2	257.3 ± 8.1	587.3 ± 4.7
26	22.3 ± 1.5	42 ± 0.0	62.7 ± 3.8	225.3 ± 12.9	449 ± 21.3
29	20 ± 1.0	37 ± 4.4	52.7 ± 4.2	191.7 ± 0.6	393.7 ± 5.7
32	17.3 ± 2.1	30.7 ± 2.3	46.7 ± 2.3	174.7 ± 10.1	361.3 ± 8.3
35	*-*	*-*	*-*	*-*	*-*

“-”: Unable to complete development.

**Table 2:** Mean (±SE) development threshold temperatures (D_0_) and thermal summation constants (K) for four development stages and the total development time of *S. nudiseta*, and the coefficient of determination (R^2^) of thermal summation models.

Development Events	K (Degree Hours)		D_0_ (°C)		R^2^
	Mean	SE	Mean	SE	
Hatching	340.14	25.52	11.81	0.91	0.98
First-ecdysis	675.59	42.10	10.19	0.87	0.98
Second-ecdysis	926.59	64.58	11.41	0.88	0.98
Pupariation	4396.68	190.65	6.38	0.79	0.96
Eclosion	7870.13	538.54	9.28	1.03	0.96

SE: Standard error.

**Table 3:** Equations, degrees of freedom (df), and coefficients of determination (R^2^) of the relationship between the body length (L) (mm) of *S. nudiseta* larvae and the time after hatching (T) (d) at five constant temperatures.

Temperature (°C)	Simulation Equation	df	R^2^
20	L = 1.58489 + 1.01858T + 0.33879T^2^ − 0.0291T^3^	33	0.997
23	L = 1.56706 + 2.09892T + 0.30901T^2^ − 0.0412T^3^	26	0.995
26	L = 1.24986 + 3.08565T + 0.22878T^2^ − 0.04961T^3^	23	0.983
29	L = 1.19314 + 4.03485T + 0.27467T^2^ − 0.08814T^3^	19	0.970
32	L = 1.47901 + 6.51375T − 0.50281T^2^ − 0.04147T^3^	15	0.995

F-values of the equations were >204.2. *p*-values were <0.001.

**Table 4:** Mean (±SD) time (h) to developmental event of *S. nudiseta* at four fluctuating temperatures.

Development Events (°C)	Hatching	First-Ecdysis	Second-Ecdysis	Pupariation	Eclosion
FT1	30.0 ± 2.0	53.3 ± 1.2	80.0 ± 0.0	266.0 ± 9.2	597.3 ± 18.1
FT2	24.0 ± 0.0	48.3 ± 0.6	69.7 ± 4.0	232.3 ± 23.5	461.7 ± 22.8
FT3	20.7 ± 0.6	40.3 ± 0.6	56.0 ± 1.7	192.0 ± 8.5	397.0 ± 13.5
FT4	18.0 ± 1.7	30.7 ± 1.2	46.3 ± 1.5	174.3 ± 14.5	*-*

FT1 (mean = 23 °C; sequential temperature phases: 20 °C, 23 °C, 26 °C), FT2 (mean = 26 °C; sequential temperature phases: 23 °C, 26 °C, 29 °C), FT3 (mean = 29 °C; sequential temperature phases:26 °C, 29 °C, 32 °C), and FT4 (mean = 32 °C; sequential temperature phases: 29 °C, 32 °C, 35 °C). “-”: Unable to complete development.

**Table 5:** Development data from different studies of *S. nudiseta*.

Origin	Site	Type of Food	Temp (°C)	TDT (D) ^a^
Velásquez et al. [10]	Alicante (IMLA, Spain)	pork liver	15.00 20.00 25.00 30.00	46.50 ± 0.97 26.33 ± 0.48 17.50 ± 0.78 15.39 ± 0.32
Rabinovich [17]	Caracas (Venezuela)	meat	20 ± 1.00 28 ± 1.00	44.9 ± 25.50 32.7 ± 16.20
Aruna Devi et al. [27]	Malaysia (USM), Penang	beef meat	26 ± 2.50	14.0 ± 1.00
Krüger et al. [26]	Curitiba (Brazil)	flour	26 ± 1.00	27.10
Kumara et al. [28]	Penang (Malaysia)	beef meat	28.5 ± 1.50	13.4 ± 0.80
Present study	HaiKou (China)	pig lunge	20.00 23.00 26.00 29.00 32.00	30.5 ± 0.29 24.5 ± 0.20 18.7 ± 0.89 16.4 ± 0.23 15.1 ± 0.35

^a^ Duration of development (days).

## Data Availability

Data will be made available on request.
